# Supplementary material for: Cross-sectional validation of the COPD Assessment Test (CAT) among chronic obstructive pulmonary disease patients in rural Uganda
Source: PLOS Glob Public Health. 2023 Jun 5;3(6):e0002013. doi: 10.1371/journal.pgph.0002013 (PMC10241354; doi:10.1371/journal.pgph.0002013)
Supplement: Attachment — Submitted filename: Response to the comments of the reviewers.pdf [file pgph.0002013.s001.pdf]

Response to the comments of the reviewers.

Reviewer #1: This study is interesting and aims to test an alternative tool for assessing the health status of COPD affecting the quality of life. Some previous published articles conducted in other countries, e.g., the UK, Japan, Thailand, and Greece, are similar to this study.

Thank you for the comment.

However, I have some questions for the authors to improve the work as follows.

Introduction: The authors mentioned the high prevalence of HIV in Sub-Saharan Africa without showing any link with COPD which is the main outcome of interest.

Thank you for the comment. The sentence has been revised to highlight the relationship between HIV, Tuberculosis and COPD, line 67- 70.

Methods (study setting): The authors only presented the setting in the area of 'Nakasake', BUT not the clinic or the LODOT-BCOPD trial which may be relevant to this study.
Thank you for the comment. A description of the clinic has been added, line 102- 107 and additional information of the health sub-district has been included. Information on the LODOT-BCOPD trial has been added under the "study population" sub- heading, line 113- 116 and a citation providing extensive information on the trial has been included.

Methods (study procedures): Participants were interviewed conveniently in either English or a local dialect – Luganda. Use of different languages might affect the authors' ability to accurately convey the meaning of the data
Thank you for the comment. This statement has been revised to "Upon completion of the informed consent process, participants were interviewed in their preferred language by the study nurse or doctor but all of them chose Luganda," line 125- 126.

Results and discussion: The authors recruited participants who were diagnosed with COPD; however, there are no details regarding the COPD staging (e.g., GOLD) which, itself, is related to the quality of life among COPD patients. Moreover, Table 1 presents participant characteristics; however, the authors did not present or discuss how these data affected the findings of the CAT/SGRQ test outcomes.

Thank you for the comment. We recognize that details on COPD staging were not included. This is because we did not collect the parameters that would support the staging. This has been included as a limitation of the study, line 213- 214. Secondly, the study did not have sufficient power to allow us to assess for the effect of participant characteristics on the CAT/ SGRQ test outcomes.

Reviewer #2: The study replicates a study of Validation of CAT in Uganda. This has been done all over the world and not necessarily novel, though it could be argued to be novel in Uganda. It is a study that validates the use of CAT scoring system for measuring quality of life and has the advantage of its simplicity for use in in clinic settings.

Thank you for the comment. We agree with you that in Uganda, the study is novel and addresses one of the hinderance clinicians face in assessing the quality of life of COPD patients.

As per the translation, it seems that the CAT and SGRQ questionnaires were not translated to the local language via standards processes of translating a questionnaire via bi-directional processes for consistency in language especially in a self-administered tool. This creates a bias in interpretation depending on the ability of the transcriber or interpreter who reads out the questions. This could potentially be an issue as per the reproducibility of this study and the findings may vary depending on the ability of the interpreter.

Thank you for the comment. A statement on how the tools were translated has been added, line 146- 150. The validated Luganda versions of the questionnaires used in this study underwent the proper steps of questionnaire translation such as back to back translation [1, 2].

[1] B. W. Morgan *et al.*, "Validation of the Saint George’s respiratory questionnaire in Uganda," *BMJ Open Respir Res,* vol. 5, no. 1, p. e000276, 2018.

[2] CAT. "The COPD Assessment Test (CAT)." https://www.catestonline.org/hcp-homepage/research.html (accessed 6th April 2023).
