# Supplementary material for: Cross-sectional validation of the COPD Assessment Test (CAT) among chronic obstructive pulmonary disease patients in rural Uganda
Source: PLOS Glob Public Health. 2023 Jun 5;3(6):e0002013. doi: 10.1371/journal.pgph.0002013 (PMC10241354; doi:10.1371/journal.pgph.0002013)
Supplement: Attachment — Submitted filename: Response to comments.pdf [file pgph.0002013.s002.pdf]

Responses to the comments.

The aim of the study was to compare and validate the CAT questionnaire against the SGRQ questionnaire. However, in lines 140 to 144, it is said that "The validated Luganda version of the CAT questionnaire..." was used – please clarify this as it sounds as if the CAT in Luganda had been validated prior to this study.

Thank you for the comment. This statement has been revised and it now reads “the official Luganda version of CAT was provided by MAPI research trust, PROVIDE, 27 rue de la Villette, 69003 Lyon, France who are authorised to provide translated versions of the CAT questionnaire upon request [33]. Prior permission was sought to use both questionnaires

Citations of previous publications should be made only when relevant. Please remove:
- reference 12 from line 70 as this paper is on asthma and allergic rhinitis, not on COPD.
- references 12, 14 and 15 from line 72 as these papers focus on asthma/allergic rhinitis, tuberculosis and malnutrition and do not provide data/findings supporting your statement on biomass or urbanisation.

Thank you for the comment. The reference list has been revised and updated.

Please revise the English, as there are several sentences where words seem to be either missing or verbs incorrectly conjugated (for example, in line 140 “The study used a validated Luganda version of the SGRQ that had undergo the….”)

Thank you for the comment. The manuscript has been revised and checked for grammatical errors.

Please amend your detailed Financial Disclosure statement. This is published with the article. It must therefore be completed in full sentences and contain the exact wording you wish to be published.

a. State the initials, alongside each funding source, of each author to receive each grant.
b. If any authors received a salary from any of your funders, please state which authors and which funders.

If you did not receive any funding for this study, please simply state: “The authors received no specific funding for this work.”

Thank you for the comment. This has been updated.

We do not publish any copyright or trademark symbols that usually accompany proprietary names, e.g., ©, ®, ™ (e.g., next to drug or reagent names). Please remove all instances of trademark/copyright symbols throughout the text, including ™ on pages 8 and 19.

Thank you for the comment. This has been updated.

References that have been updated:

Removed

(3) J. L. López-Campos, W. Tan, and J. B. Soriano, "Global burden of COPD," vol. 21, no. 1, pp. 14-23, 2016, doi: https://doi.org/10.1111/resp.12660. This reference has been removed and replaced.

(5) M. Varmaghani, M. Dehghani, E. Heidari, F. Sharifi, S. S. Moghaddam, and F. Farzadfar, "Global prevalence of chronic obstructive pulmonary disease: systematic review and meta-analysis," (in eng), no. 1687-1634 (Electronic). This reference has been removed.

(12) B. W. Morgan *et al.*, "Asthma and Allergic Disorders in Uganda: A Population-Based Study Across Urban and Rural Settings," (in eng), *J Allergy Clin Immunol Pract*, vol. 6, no. 5, pp. 1580-1587.e2, 2018 Sep - Oct 2018, doi: 10.1016/j.jaip.2017.11.032. This reference has been removed.

(14) B. Mete, E. Pehlivan, G. Gülbaş, and H. Günen, "Prevalence of malnutrition in COPD and its relationship with the parameters related to disease severity," (in eng), *International journal of chronic obstructive pulmonary disease*, vol. 13, pp. 3307-3312, 2018, doi: 10.2147/COPD.S179609. This reference has been removed.

(15) H. I. Yakar, H. Gunen, E. Pehlivan, and S. Aydogan, "The role of tuberculosis in COPD," (in eng), *International journal of chronic obstructive pulmonary disease*, vol. 12, pp. 323-329, 2017, doi: 10.2147/COPD.S116086. This reference has been removed.

(20) B. W. Morgan *et al.*, "Validation of the Saint George's Respiratory Questionnaire in Uganda," (in eng), *BMJ Open Respir Res*, vol. 5, no. 1, p. e000276, 2018, doi: 10.1136/bmjresp-2018-000276. This was repeated and removed.

Added

C. D. Mathers and D. Loncar, "Projections of global mortality and burden of disease from 2002 to 2030," *PLoS medicine*, vol. 3, no. 11, p. e442, 200. This replaced Reference 3.
